# Structurally diverse acylsugars on wild tomatillo fruit surfaces

**DOI:** 10.1093/hr/uhag177

**Published:** 2026-04-28

**Authors:** Lillian Nowack, Rocio Deanna, Stacey D Smith, Craig A Schenck

**Affiliations:** Department of Biochemistry, University of Missouri, Columbia, MO, USA; Interdisciplinary Plant Group, University of Missouri, Columbia, MO, USA; Botany and Mycology Unit, Finnish Museum of Natural History & Organismal and Evolutionary Biology Research Programme, University of Helsinki, Helsinki, Finland; Museo Botánico de Córdoba (IMBIV-CONICET-UNC), Córdoba, Argentina; Department of Ecology and Evolutionary Biology, University of Colorado, Boulder, CO 80309, USA; Department of Biochemistry, University of Missouri, Columbia, MO, USA; Interdisciplinary Plant Group, University of Missouri, Columbia, MO, USA

## Abstract

Plants have evolved a structurally diverse chemical repertoire to mediate various environmental interactions. Yet, little is known about the chemical complexity and metabolic pathways across many plant genera. Acylsugars are a class of specialized metabolite widely distributed across the Solanaceae that play a role in deterring herbivores, insects, and fungal pathogens. Although acylsugars have been extensively characterized from leaf glandular trichomes, they have also been detected in other organs. Nevertheless, the biosynthesis, structures, and function of acylsugars outside of leaf trichomes remain unknown. Here, we performed organ-specific metabolomics across 28 *Physalis* species, an emerging model and crop genus closely related to important *Solanum* crops. Automated feature extraction revealed thousands of mass spectral features across the different species and organs. Acylsugar mass spectral features were manually annotated from surface extracts, revealing at least 323 unique acylsugars, substantially expanding the known acylsugar diversity in the *Physalis* genus. Some, but not all, species in the *Physalis* genus accumulated acylsugars on the fruit surface, and acylsugars were as abundant or sometimes more abundant in fruits than in leaves or calyces. Hierarchical clustering and phylogenetic tests indicated that species with similar acylsugar profiles did not cluster taxonomically. To determine the biochemical mechanism underlying acylsugar structural diversity, we characterized the first step of acylsugar biosynthesis, catalyzed by an acylsugar acyltransferase. Three *Physalis* ASAT1 homologs displayed broad substrate preferences, which may partially explain the differences in acylsugar profiles. The diverse fruit-localized acylsugars across the *Physalis* genus can inform engineering strategies for increased crop resilience.

## Introduction

Plants produce a repertoire of structurally diverse metabolites, generally divided into two classes—core and specialized (also known as secondary metabolites or natural products). Specialized metabolites largely function in abiotic and biotic stress response [2–5] and exhibit greater structural diversity than core metabolites. These specialized metabolites are downstream of core metabolic pathways and have arisen from the co-option of duplicated core metabolic enzymes [6–8]. Unlike core metabolism that is conserved across plants, specialized metabolic pathways are typically lineage and tissue/organ-specific [3, 9, 10]. While the ecological significance of specialized metabolites is increasingly recognized, many biosynthetic pathways and the downstream chemical diversity remain largely unexplored, particularly in non-model plants. This knowledge gap limits our ability to leverage biologically active metabolites for crop enhancement, stress resistance, and to co-opt these metabolites for medicinal and industrial purposes.

The Solanaceae family, which includes economically important species, such as tomato, eggplant, potato, and petunia, is well known for producing a diverse array of specialized metabolites, including terpenes, alkaloids, phenylpropanoids, and acylsugars [11]. Acylsugars usually accumulate in glandular trichomes present on leaf and stem surfaces, and function in defense against insects, herbivores, and microbial pathogens, and contribute to reducing the impact of abiotic stress [12–17]. The mechanism of action of acylsugars remains unclear; however, trichome exudates are sticky and immobilize or suffocate insects on the leaf surface and acylsugars function similarly to waxes to prevent desiccation [18]. They are composed of a sugar core, usually sucrose or glucose, and acyl chains of various lengths and branching patterns that are attached at specific hydroxyl groups on the sugar core [19]. Acylsugar chemical diversity has been investigated at various taxonomic levels across the Solanaceae, including the genera *Nicotiana* and *Solanum* [20, 21], as well as the whole family [22]. One major theme from trichome-localized acylsugar profiling across a broad range of Solanaceae is despite being assembled from simple precursors, acylsugar structural diversity is extensive. The key steps of acylsugar biosynthesis, esterification of an acyl-CoA to the sugar core, are catalyzed by a set of acylsugar acyltransferases (ASATs) that are highly expressed in the trichomes and have been biochemically characterized from many species in the Solanaceae family [6, 22–25].

Although acylsugars are most prevalent in glandular trichomes on vegetative tissue, they have been detected in other organs, including roots in tomato and fruits in some *Physalis* species. [26, 27]. With roughly 90 species [28], *Physalis* is one of the largest genera in the Solanaceae and falls within the large berry clade, Solanoideae, that also includes tomatoes, peppers, and eggplants [29]. Species of *Physalis* are gaining importance as both food crops and model organisms [30–32]. The *Physalis* genus includes the crop tomatillo (*Physalis philadelphica* Lam.) in addition to goldenberry (*Physalis peruviana* L.), groundcherries (*Physalis grisea* (Waterf.) M. Martínez and *Physalis pruinosa* L.), and other species with edible fruits, medicinal properties, or grown as ornamentals [30, 33]. Mexico is the largest producer of tomatillo with an export of approximately 698000 tons valued at 66.6 million USD in 2016 [33]. Although a few acylsugar structures have been NMR characterized from fruit or calyx of specific species in the *Physalis* genus [34–37], the biological functions and distribution of fruit-localized acylsugars across the genus are unknown.

Although acylsugars are best studied in vegetative organs, their presence on the fruit surface suggests they may play novel roles in defense (i.e., protecting developing seeds). We hypothesize that fruit-surface localized acylsugars are synthesized and secreted independently from glandular trichome biosynthesis of leaf acylsugars. To gain insight into the distribution of fruit-surface localized acylsugars, we characterized acylsugar chemical diversity from leaf, calyx, and fruit surfaces across the *Physalis* genus. To determine similarities or differences of fruit-localized acylsugar biosynthesis, we characterized three *Physalis* ASAT1 homologs. Our findings provide valuable insights into the distribution and biosynthesis of fruit-surface localized acylsugars across *Physalis* and lay the groundwork for future studies investigating their biological function.

## Results

### Metabolic diversity present in surface extracts of *Physalis* and related species

To characterize the metabolic landscape across the *Physalis* genus*,* we extracted surface metabolites from three organs, leaf, calyx, and fruit, and performed metabolomics using LC–MS. Surface extracts were prepared from 28 *Physalis* species and nine outgroup species. Some of these samples were grown from seed in growth chambers, while the majority came from extracts performed in the field in central Mexico and surrounding regions, thus not all plants had complementary leaf, calyx, and fruit samples given the nature of sample collection (Supplementary Table S1). Extracts were analyzed using UHPLC–MS primarily in negative ion mode. Mass spectral features were identified using MSDial [38] across all extracts with limited processing to attempt to capture as many unique mass spectral features as possible (see Methods). The number of mass spectral features identified in each extract varied widely. Some extracts had as few as 500 unique mass spectral features and as much as 8000 (Supplementary Fig. S1). To assess overall metabolic diversity across organs and species, we calculated Shannon entropy values based on the presence or absence of all mass spectral features identified. *Physalis viscosa* L. had the highest Shannon entropy value, whereas *Withania riebeckii* Balf.f. had the lowest, generally consistent with the numbers of mass spectral features identified, and peaks observed in chromatograms (Supplementary Fig. S2a). To determine if there were organ-specific differences in metabolic complexity, we averaged leaf, calyx, and fruit Shannon entropy values across all species. Shannon entropy values for calyx and fruit were not significantly different, suggesting that their average metabolic complexity is comparable across the species analyzed (Supplementary Fig. S2b). However, leaf metabolic complexity was significantly lower than both fruit and calyx (Supplementary Fig. S2b).

### Manual annotation reveals tremendous acylsugar variation across *Physalis*

Although thousands of mass spectral features were detected across *Physalis* species, the major challenge with large scale metabolomics is confident mass spectral feature annotation (Supplementary Figs S1). Based on the mass-to-charge (*m/z*) values and fragmentation patterns, many of these mass spectral features were hypothesized to be acylsugars, which are well studied within the Solanaceae family. Acylsugar mass spectral features were manually annotated in negative ion mode using the intact formate adduct mass and fragmentation patterns (Supplementary Fig. S3a and b), yielding information about the lengths and number of acyl chains. To determine if replicate extracts had similar acylsugar types and abundances, we initially ran biological replicate injections from a few species. Acylsugar annotations and normalized peak areas from replicate injections were comparable, which provided confidence to run a single injection from all plant extracts from different organs (Supplementary Fig. S4).

Manual annotation of acylsugars revealed 323 unique acylsugar types across organs and species. (Supplementary Table S2). Acylsugars with two acylations (diacylsugars) represented 9% of the total annotated structures, whereas acylsugars with three (triacylated) and four (tetraacylated) acylations represented 63.8% and 27.2%, respectively (Supplementary Table S2, Supplementary Fig. S5), suggesting that there is a biochemical bias towards the accumulation of triacylated and tetraacylated acylsugars. On a species level, accumulation patterns of diacylsugars, triacylsugars, and tetraacylsugars was more variable (Supplementary Fig. S5). For example, *Eriolarynx lorentzii* (Dammer) Hunz. calyx accumulated 66.7% tetraacylated acylsugars (Supplementary Fig. S5). *Physalis aggregata* Waterf*.,* on the other hand, accumulated 2 diacylated, 17 triacylated, and 4 tetraacylated acylsugars, consistent with the overall acylation trends observed at the genus level (Supplementary Fig. S5). In contrast, many of the 323 manually annotated acylsugars had identical annotations but differing retention times (RTs), indicating the presence of isomers differing in either acyl chain positioning on different hydroxyl groups or acyl chain branching pattern. Two annotations, which had numerous isomers, S3:21(4, 5, 12) and S3:22(5, 12), where S indicates sugar core (sucrose in this case) with three acylations: totaling 20 and 21 carbons, respectively and with the individual acyl chain lengths of 4, 4, 12 and 4, 5, and 12 carbons each. S3:21(4, 5, 12) had 14 distinct RTs in 19 different species and S3:22(5, 12) had 15 distinct RTs in 17 different species (Supplementary Table S2).

To complement the negative mode annotations, we performed MS/MS on a surface extraction from *Physalis nicandroides* Schltdl. in negative mode and positive mode MS analysis for a subset of samples (Supplementary Figs S3c and S6, Supplementary Table S2). MS/MS analysis was performed for the five most abundant acylsugars from *P. nicandroides* with *m/z* of 681.34, 695.35, 723.36, 737.37, and 751.38. Acylsugar annotations of the MS/MS fragmentation patterns were consistent with MS annotations (Supplementary Figs S6 and S7, Supplementary Table S2). Positive mode annotations generally confirmed negative mode annotations and provided evidence for locations of acyl chains on the pyranose (six-membered) or furanose (five-membered) ring. Positive mode annotations revealed that the long acyl chain, usually 10 or 12 carbons long, was attached to the pyranose ring of the sugar core (Supplementary Table S2, Supplementary Fig. S3c). The acyl chain attachment was consistent with previously elucidated NMR structures of *Physalis* acylsugars [36, 37]. However, the short and medium chains were more variable on whether they were attached to the pyranose or furanose ring of the sugar core, possibly suggesting enzymatic flexibility for other steps in the pathway.

Although fragmentation patterns yielded ions consistent with a disaccharide sugar core lacking acyl chains in many extracts, the identity of the sugar core was not confirmed from LC–MS fragmentation patterns alone. Based on previously characterized NMR structures from species in the *Physalis* genus, we hypothesized that the disaccharide core present across most of the *Physalis* genus is sucrose [27, 34–37]. To confirm the sugar core type, acylsugars were extracted from a subset of species (*P. philadelphica, Physalis hederifolia* A. Gray*, Physalis heterophylla* Nees), the acyl chains removed, then the remaining sugar core was derivatized and injected into GC–MS. *Solanum pennellii* Correll was used as a positive control, as it has been shown to produce both acylglucoses and acylsucroses [39]. Our analysis confirmed the presence of both glucose and sucrose cores in *S. pennellii* Correll acylsugars (Supplementary Fig. S8). Only sucrose was detected in *P. philadelphica,* consistent with LC–MS acylsugar annotations (Supplementary Table S2). Based on LC–MS annotations, *P. hederifolia, P. heterophylla* produce primarily acylsucroses. GC–MS analysis of *P. hederifolia, P. heterophylla* cores reveal predominant sucrose and minor glucose peaks (Supplementary Fig. S8).

Across the *Physalis* genus, species produced acylsugar chains with as few as 2 carbons and up to 12, with most acylsugar structures consisting of at least one medium-chain acyl chain (C8–C12), and several short chains (C2–C6) (Supplementary Table S2, Supplementary Fig. S9). LC–MS fragmentation pattern does not provide information about branching patterns of acyl chains or locations of potential double bonds. To provide additional structural information about acylsugar acyl chains, GC–MS analysis was performed on a subset of species with abundant acylsugars. GC–MS revealed the presence of several branched chains such as isobutyryl (iC4), isovaleryl (iC5), and isohexanoyl (iC6) (Supplementary Fig. S10), which arise from branched-chain amino acid precursors valine, isoleucine, and leucine [40]. Detectable levels of desaturated C5 were also present in these species, but generally in low amounts. The longer acyl chains (C8–C12) were typically straight and unsaturated (Supplementary Fig. S10), consistent with previously elucidated NMR structures [34–37]. Various complementary analytical approaches confirm the presence of a large degree of acylsugar structural variation across the *Physalis* genus*.*

### Organ-specific variation in acylsugar accumulation

**Figure 1:**
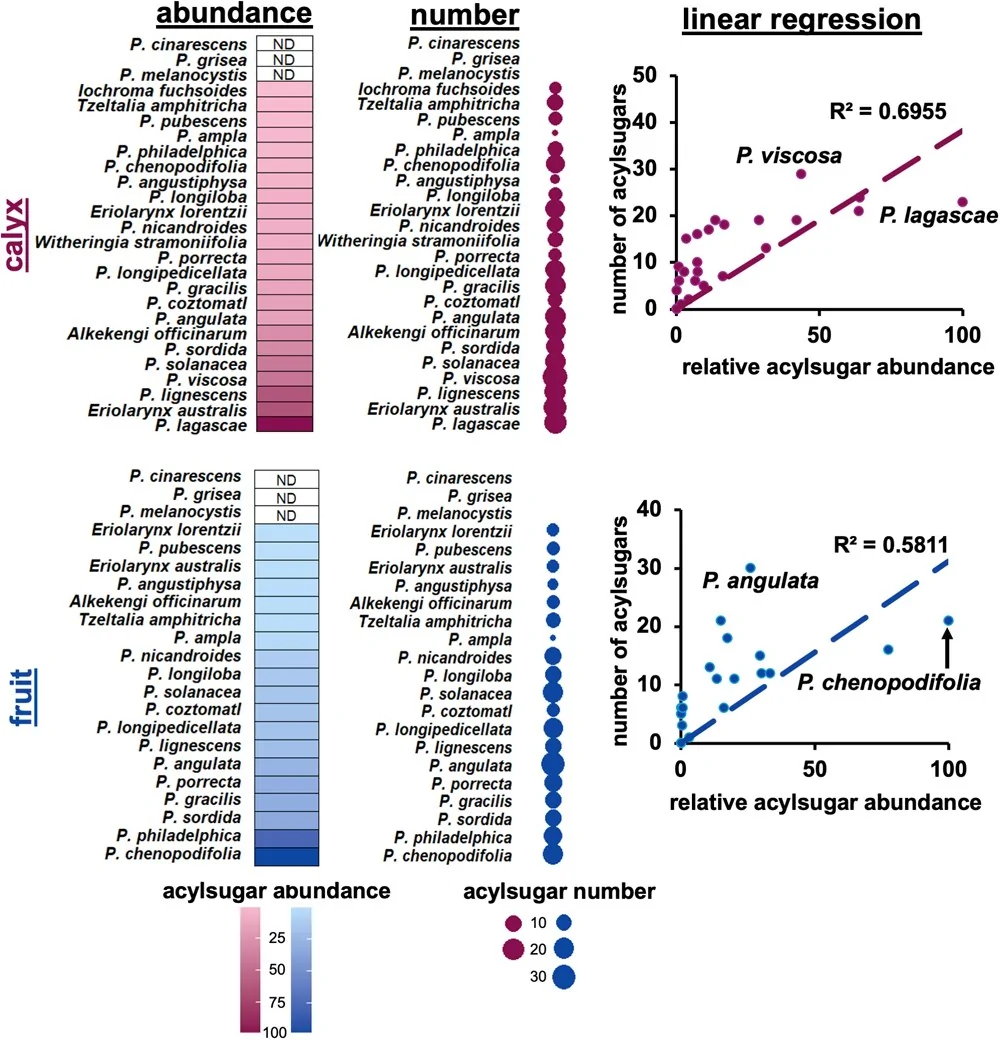
Correlation of number and abundance of fruit and calyx acylsugars from the *Physalis* genus. Acylsugars were quantified using the total peak area of all annotated acylsugars corrected for internal standard peak area and sample dry weight. In an organ specific manner, the most abundant extract was set to 100% and all other extracts were normalized to this value. N.D., below detection limit. Size of circle indicates number of acylsugar types in calyx and fruit. Lack of circle indicates no detectable acylsugars from that organ. Linear regression of the relationship between relative abundance and number of acylsugars, with *R*^2^ values for line of best fit shown.

Although a handful of acylsugar structures have been NMR characterized from fruit of some *Physalis* species [27, 36], we wanted to test if all the *Physalis* genus accumulates acylsugars on the fruit surface. To evaluate organ-specific acylsugar differences, we compared the abundance and number of acylsugars in calyx and fruit across a subset of *Physalis* species (Fig. 1). Fruit localized acylsugars were detected from 23 species, whereas five species had no detectable fruit localized acylsugars (Supplementary Fig. S11). While abundant acylsugars were detected on the fruit and calyx in many species, they were not detectable from leaf extracts of most *Physalis* species profiled, except for *P. pruinosa, P. heterophylla,* and *P. hederifolia* (Supplementary Table S2, Supplementary Figs S11 and S12)*.* This is supported by glandular trichome counts, as most species had few to no glandular trichomes on the leaf surfaces used for acylsugar extractions (Supplementary Fig. S13a). Both acylsugar abundance and number varied widely across species and organ types (Fig. 1). *Physalis ampla* Waterf. produced only one annotatable acylsugar on the fruit; however, *Physalis angulata* produced more than 30 (Fig. 1). Generally, acylsugar profiles were similar in calyx and fruit, with fruit producing a larger number of annotatable acylsugars, possibly due to fruit extracts having more abundant acylsugars (Supplementary Fig. S6). To test if calyx acylsugar abundance is correlated with fruit acylsugar abundance, we performed a regression analysis between fruit and calyx acylsugar traits. We find a lack of correlation between these two traits, suggesting independent control over fruit and calyx localized acylsugar accumulation (Supplementary Fig. S14a). A parallel analysis incorporating phylogenetic effects based on the molecular phylogeny of Deanna et al. [1] also failed to recover a relationship between fruit and calyx acylsugar abundance across species (Supplementary Fig. S14b). While many species had abundant acylsugars on the fruit surface, it remains unclear whether the acylsugars are being biosynthesized on the fruit surface, the calyx, or both.

To assess the relationship between the number and abundance of acylsugars, linear regression was performed with the normalized abundance values and number of acylsugars for all samples in an organ-specific manner. The coefficients of determination (*R*^2^) values for calyx and fruit were 0.6955 and 0.5811, respectively (Fig. 1), indicating a moderately positive correlation between the abundance and number of acylsugars. Some species deviated from this trend. For example, *P. philadelphica* fruit displayed a high abundance but less in regard to number, while others, such as *P. viscosa* calyx, showed the inverse. These patterns suggest that while abundance is influenced by the number of acylsugars produced, there are other contributing factors.

### Acylsugar structural diversity in relation to the phylogenetic history

**Figure 2:**
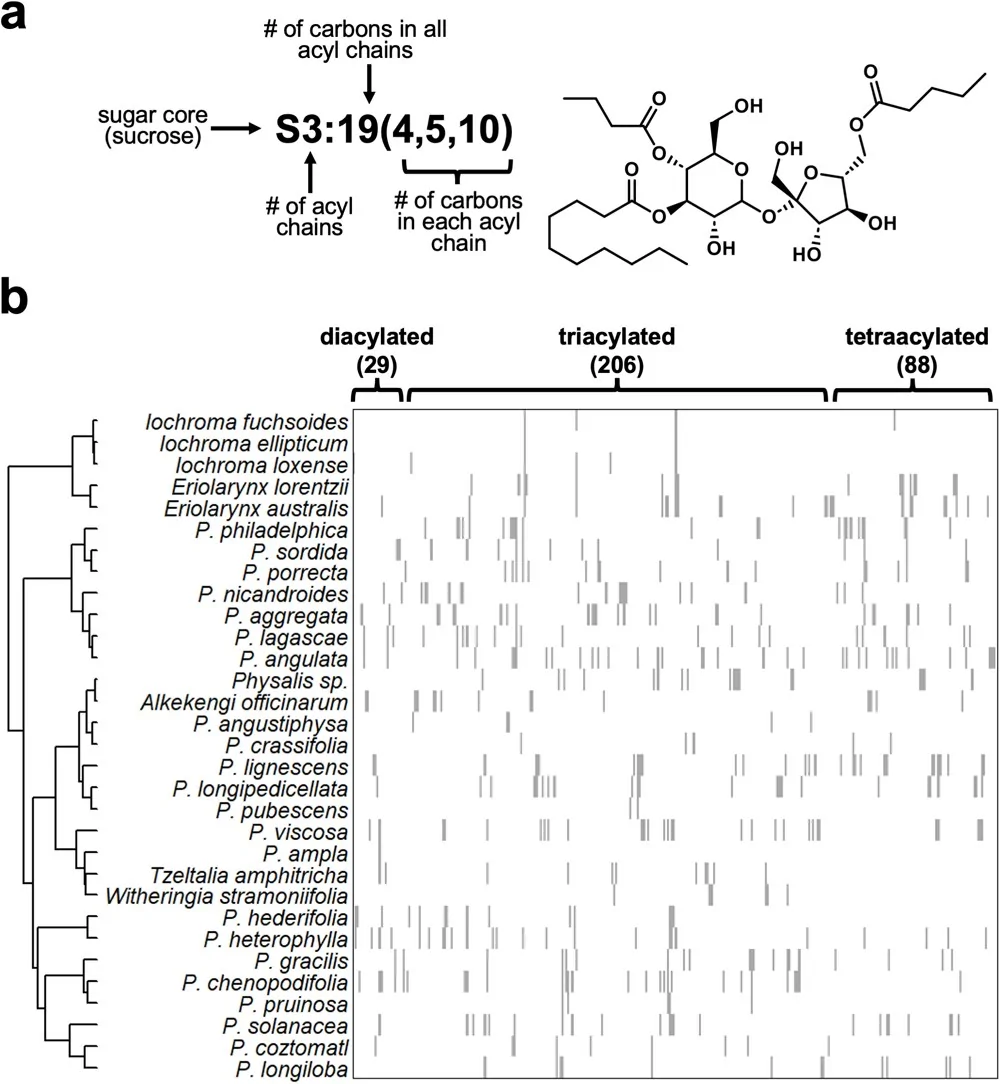
Hierarchical clustering of acylsugar structural variation across the *Physalis* genus and other closely related genera. (a) Acylsugar nomenclature used in this and other studies with a representative triacylated structure shown. (b) HCA of presence/absence of each annotated acylsugar. Each row represents a species, while each column represents a single acylsugar annotation in order of number of acylations, then increasing size in total number of carbons in the acyl chains. Number of diacylated, triacylated, and tetraacylated acylsugars identified are shown in parenthesis. Grey indicates presence of an acylsugar, white indicates absence. Acylsugar presence for each species represents the sum of all acylsugar types present across all tissues.

Next, we wanted to examine the degree to which acylsugar production relates to phylogenetic relationships among the species. To quantify the similarity of acylsugar profiles across species and organs, we first carried out hierarchical clustering analysis (HCA) and principal coordinate analysis (PCoA) based on the absence and presence of each of the 323 manually annotated acylsugars. Out of the 323 annotated acylsugars, 233 (~72%) were detected in only one species, highlighting the predominance of species-specific acylsugars (Fig. 2). Furthermore, only 39 were shared across three or more species. This indicates limited overlap in acylsugar production across the genus, despite their close evolutionary relationship. Moreover, the patterns of clustering recovered from HCA (Fig. 2b) were only loosely tied to the taxonomic groupings based on the most recent phylogeny of Physalideae [1]. In particular, some of the outgroup taxa (*Eriolarynx* (Hunz.) Hunz., *Iochroma* Benth.) did cluster separately from the rest of the species (Fig. 2b), but others appear very similar to some *Physalis* species. To further explore acylsugar variation, PCoA was conducted using the same binary acylsugar matrix. Similar to HCA, the PCoA highlighted distinct clusters of species that have similar acylsugar profiles (Supplementary Fig. S15). Here again, the samples from the *Iochroma* and *Eriolarynx* genera appear very distinct from the rest, while the *Witheringia* L'Hér. and *Tzeltalia* E. Estrada & M. Martínez genera grouped with species from the *Physalis* genus.

To more rigorously assess the relationship between chemical profiles and the phylogeny, we computed two measures of phylogenetic structure in the acylsugar data and tested for significant signal. First, we calculated Blomberg's K [41] for acylsugar abundance in fruits and calyces using a sample of 100 Bayesian trees from the phylogenetic analysis of [1] to account for phylogenetic uncertainty. Values of K ranged from 0.16 to 0.64 across trees and traits, and none were significantly different from zero (Supplementary Table S3), suggesting no detectable phylogenetic signal. We found a similar lack of signal in the type of acylation. We scored each of the species as present or absent for each class of acylsugars (di, tri, tetra) in any organ and used Fritz and Purvis's D statistic [42] to test for signal in these binary variables. Again, using the sample of 100 trees, these three traits presented values of D closer to 1, which is the expected value from no phylogenetic structure (Supplementary Table S4). It is worth noting, however, that many of the sampled species are not included in the phylogeny (e.g., only 18 species are present in the phylogeny and have calyx acylsugar abundance data), and generally the power to detect signal is low with fewer than 20 species [41].

### Identification and biochemical characterization of *Physalis* ASAT1s

**Figure 3:**
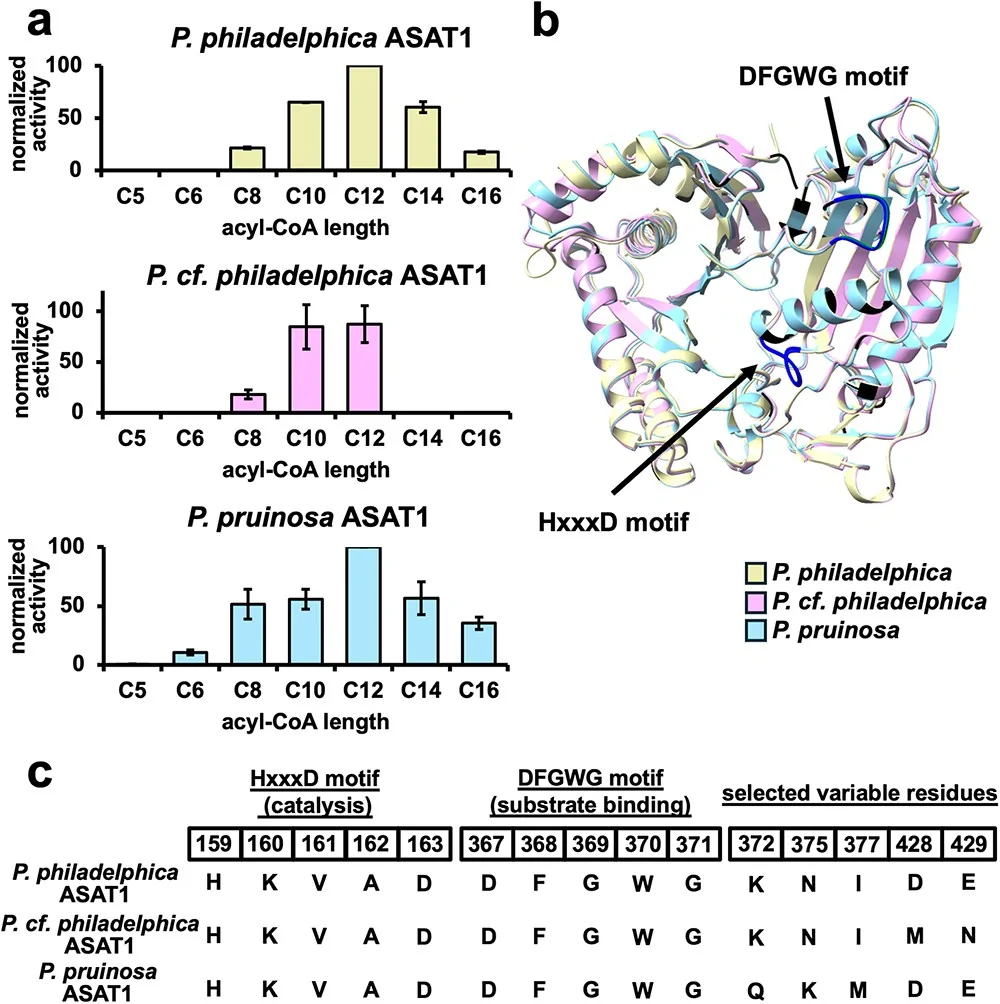
*Physalis* ASAT1 *in vitro* biochemical and structural characterization. (a) Substrate promiscuity of *Physalis* genus ASAT1s. Enzyme activity was tested with purified recombinant enzymes with varying lengths of acyl-CoA (250 μM) and sucrose (2 mM) and monitored for production of monoacylated products using LC–MS. Values are means ± standard deviation of two biological replicates. (b) Structural model overlays of ASAT1s, with amino acid changes in black, and arrows point to catalytic motifs (dark blue). (c) Amino acid alignment of conserved and variable regions of ASAT1s. Amino acid numbering based on *P. pruinosa* ASAT1.

To investigate the biochemical basis of acylsugar chemical diversity, we identified and characterized three ASAT1 homologs from different *Physalis* : *P. pruinosa, P. philadelphica,* and *P. cf. philadelphica.* ASAT1 candidate genes were identified using biochemically characterized *Solanum lycopersicum* L. ASATs to BLAST the *P. pruinosa* genome. BLAST hits were filtered based on the presence of two conserved domains present in BAHD acyltransferases, HxxxD and DFGWG motifs. A phylogenetic analysis was performed with *P. pruinosa* candidates together with biochemically characterized ASATs from Solanaceae species (Supplementary Fig. S16). One *P. pruinosa* candidate grouped within a clade containing previously characterized ASAT1s called ancestral ASAT1s (AncASAT1s) as they are present in most Solanaceae lineages but absent in *Solanum* (Supplementary Fig. S16). Primers designed for *P. pruinosa* ASAT1 were used to amplify and clone *P. pruinosa* ASAT1, as well as homologous ASAT1 sequences from *P. philadelphica* and *P. cf. philadelphica* ASAT1. Substrate specificity of recombinantly expressed and purified ASAT1s was assessed *in vitro* using commercially available acyl-CoAs of varying lengths together with sucrose. *In vitro* assays revealed slight differences in substrate specificity. While all three ASAT1s preferred C12 CoA, *P. cf. philadelphica* had a narrower substrate specificity range than the other two ASAT1 homologs (Fig. 3a). The preference for the C12 product is consistent with the widespread presence of long-chain acylsugars (Supplementary Fig. S9), as well as with the presence of a long chain in most NMR elucidated structures [34–37].

To explore the biochemical basis for changes in substrate specificity, AlphaFold structural models were generated for the ASAT1s identified from the *Physalis* genus. The ASAT1s showed sequence variation, with 12 amino acid changes between *P. cf. philadelphica* ASAT1 and *P. philadelphica* ASAT1, 28 between *P. pruinosa* ASAT1 and *P. cf. philadelphica* ASAT1, and 30 between *P. pruinosa* ASAT1 and *P. philadelphica* ASAT (Supplementary Fig. S17). Structural modeling revealed slight changes in the tertiary structure of these enzymes, primarily at the N-terminus, and slight changes in the positioning of alpha-helices (Fig. 3b). Several of these changes are near conserved HxxxD and DFGWG binding motifs, necessary for catalysis and structural stability of BAHD family acyltransferases [43] and may be responsible for the substrate specificity differences observed across the ASAT1s identified in the *Physalis* genus (Fig. 3c).

## Discussion

### Metabolic diversity in *Physalis* species

Metabolic profiling across the *Physalis* genus revealed a large number of mass spectral features across different organs, with the fruit and calyx having more metabolic diversity than the leaf (Supplementary Fig. S2). Manual annotation of acylsugars reveals a large number of acylsugar types highly abundant on the fruit surface (Fig. 1, Supplementary Table S2). Differences in the amounts and types of putative metabolites identified between species and organs likely reflect genotypic differences between species, but metabolic differences are also associated with environmental factors and technical limitations with sampling and LC–MS capabilities. Plants within the Physalideae tribe, including *Physalis,* are well known for a unique morphological trait—an inflated calyx surrounding the fruit—that has been hypothesized to serve a number of ecological and physiological functions, including stabilizing the microclimate during fruit development [44], aiding in dispersal [45], and protecting the berry [46]. The presence of acylsugars localized to the fruit surface and morphological innovations suggests the presence of a multilayered defense response in *Physalis* species.

### Acylsugar diversity and biological activity implications

Acylsugars in the *Physalis* genus displayed extensive structural diversity. Over 300 distinct acylsugars were annotated, of which only ~90 were present in more than one species, showing that the majority of acylsugar structures are species-specific (Supplementary Table S2, Supplementary Fig. S2). Because of technical limitations in detection limits and separation of metabolites, the number of acylsugars across the *Physalis* genus is likely much greater than what can be annotated from LC–MS experiments. It remains unclear as to why so many distinct acylsugar compounds accumulate across the *Physalis* genus and the Solanaceae family. Producing a repertoire of acylsugar types may provide a broad layer of defense that is challenging to evolve an avoidance mechanism. Acylsugars have demonstrated roles in biotic and abiotic stress response [16] and some acylsugars with long chains have been shown to have a greater effect on certain insects [47]. Thus, different acylsugar types may have specific functions (e.g., some may be more useful in abiotic stress tolerance and others for biotic stresses).

### ASAT promiscuity enables acylsugar structural variation

Despite the evolutionary relationships among the sampled species, there are many differences in the overall acylsugar profile, abundance, and organ-specificity. *Physalis* ASAT1s grouped within a clade containing AncASAT1s from most Solanaceae lineages excluding the *Solanum* genus ASAT1s (Supplementary Fig. S16). Although AncASAT1s and *Solanum* ASAT1s acylate at different hydroxyl groups they both have broad substrate specificity profiles for the acyl-CoA substrate [21–23]. Acylsugar structural diversity reflects not only the substrate promiscuity of ASATs but also the availability of acyl-CoA substrates at the location of biosynthesis among other factors [21, 48]. This is highlighted especially by the substrate specificity and slight structural changes in the first step of the acylsugar biosynthetic pathway, catalyzed by *Physalis* ASAT1s (Fig. 3). While all *Physalis* genus ASAT1s profiled preferred long-chain acyl-CoAs, the broad substrate profile observed in *Physalis* ASAT1s (Fig. 3) may contribute to the diverse acylsugar structures observed. However, other factors, such as substrate availability, downstream biosynthetic steps, and other modifying acylsugar enzymes, such as hydrolases and invertases, also contribute to the vast number of acylsugars observed across the Solanaceae family and likely the *Physalis* genus [49, 50]. Sequence alignments and structural modeling highlight some amino acids that could play a role in the range of substrate preferences observed across *Physalis* ASAT1s (Fig. 3, Supplementary Fig. S17), which could be investigated using site directed mutagenesis. Given the broad acyl-CoA substrate profiles typical of ASATs, it could be possible to engineer ASATs for greater specificity to generate acylsugar types with increased activities.

### Acylsugars vary independently of the phylogeny

Although we are only just beginning to understand the diversity of acylsugar production in the *Physalis* genus, our results suggest that this diversity is not strongly predicted by evolutionary history. Except for species belonging to the Iochrominae subtribe (*Eriolarynx* and *Iochroma*), our clustering analyses based on chemical profiles group species together that are not closely related on the phylogeny (Fig. 2, Supplementary Tables S3 and S4, Supplementary Fig. S15). This is partly because most species accumulate distinct, species-specific acylsugar types, with only a handful of compounds shared across species (Fig. 2). In addition, when we directly estimate phylogenetic signal in acylsugar abundance and type, we find no significant phylogenetic signal, i.e., tendency for phylogenetic relationships to predict trait values (Supplementary Tables S3 and S4). While these results suggest that acylsugar profiles are not a good indicator of relatedness, our taxon sampling covers only a quarter of the species diversity within the *Physalis* genus [28]. A more resolved and comprehensive phylogeny of the *Physalis* genus together with additional acylsugar profiling from a greater number of *Physalis* species would provide a clearer picture of evolutionary trends in acylsugar production and allow us to more confidently estimate the tempo and mode of changes in acylsugar traits, such as acyl chain lengths, core types, and organ-specific accumulation.

### Widespread fruit-localized acylsugars in *Physalis*

Canonically, acylsugars are synthesized in the glandular trichomes on leaf and stem surfaces [19]. In most *Physalis* species screened, there were no detectable acylsugars in leaves (Supplementary Fig. S12), supported by few to no glandular trichomes on the leaf surface (Supplementary Fig. S13a). However, we identified many species producing fruit-localized acylsugars, a unique observation especially considering the lack of observable canonical type IV glandular trichomes on the fruit surface (Supplementary Fig. S13b). Fruit-localized acylsugars were identified in 23 of the 37 species profiled (Supplementary Fig. S11), highlighting how widespread this trait is within the genus. In many cases, acylsugars were more abundant on the fruit-surface as compared to the other organs screened, suggesting the novel localization serves an important function. Fruit-surface localized acylsugars are sticky, like trichome-localized acylsugars, and thus could serve as a trap for small insects but additionally could serve to protect the fruit from desiccation and the developing seeds from biotic stressors. Moreover, acylsugar production in fruits appears to evolve independently from production in these other organs (Supplementary Fig. S14), suggesting organ-specific transcriptional mechanisms that could be leveraged in the future for targeted crop improvements.

Despite widespread distribution of fruit-localized acylsugars across *Physalis* species, it is unknown how they are deposited on the fruit surface. Understanding the biosynthetic pathway will enable the investigation of ASAT gene expression patterns across different organs. It is possible that ASATs are expressed in modified trichomes or secretory gland cells on the fruit surface, analogous to how volatile compounds are secreted from citrus fruit peels [51, 52]. Further work is required to understand the biosynthesis and deposition of fruit-surface localized acylsugars. This knowledge can be used to remove the stickiness from fruits if it is not required for protection, or this trait could be engineered in other berry crops, like tomato, for enhanced protection.

## Materials and methods

### Field collections and plant growth

Extractions from plants in the field were performed during collection trips in central and Southern Mexico and central Argentina, except *W. riebeckii*, which was collected at the Kaisaniemi Botanic Garden (Helsinki, Finland). Plants were identified by R. Deanna, P. Zamora-Tavares, A. Herrera, O. Vargas-Ponce, and M Martínez, and voucher specimens are stored at the CORD, H, IBUG, and QMEX herbaria. Voucher information for species used in this study can be found in Supplementary Table S1.

When extractions were not performed in the field, we cultivated the plants in the greenhouse. *Physalis* seeds were obtained from the US Department of Agriculture Germplasm Resources Information Network (GRIN) or from previous collection trips. Seeds were treated with 10% sodium phosphate tribasic and 50% bleach (v/v) solution for 5 minutes with constant rocking. Seeds were rinsed five times with distilled water, placed on Whatman filter paper, and germinated at 28°C in the dark. Seedlings were transplanted to soil and grown at 27°C during the day and 22°C during the night under a 18/6 day/night cycle with a light intensity of ~9000 lux. Plants were fertilized once per week with Miracle-Gro Water Soluble All Purpose Plant Food (The Scotts Company LLC, Marysville, OH, USA). See Supplementary Table S1 for details about source, location, organ, and other details about plants used for metabolite extractions.

### Surface metabolite extraction, quantification, and detection

Metabolites used for LC–MS analysis were extracted from young leaf tissue or mature fruit and calyx using 3:3:2 isopropanol:acetonitrile:water with 0.1% formic acid [16]. Plant material was submerged in 1 ml of extraction solvent and gently agitated for 3 to 5 minutes. The extraction solvent was dried to completion using a centrivap (ThermoScientific) then resuspended in 100 μl of extraction solvent with a final concentration of 3-μM telmisartan internal standard for relative quantification. Telmisartan has a similar RT to acylsugars and is ionizable in both positive and negative modes, making it an ideal internal standard for relative quantification of acylsugars [39]. Extracts were placed in a 2 ml glass vial with an insert, then 4 μl of the extracts were injected into a Bruker Compact LC–MS qTOF with an Ascentis Express 90 Å C18 column for reverse-phase separation with the column temperature set at 30°C. For the LC gradient, the starting concentration was 95% solvent A (10 mM ammonium formate, adjusted to pH 2.8 with formic acid) and 5% solvent B (100% acetonitrile) with flow rate set to 0.3 ml/min. Metabolites were separated on a C18 reverse-phase column using a 20-minute LC gradient (Supplementary Fig. S18) using an UltiMate 3000 system operating in positive and negative ion modes.

Putative acylsugars were identified that had an RT between 6.0 and 15.5 minutes, *m/z* between 639 and 835, and adducts of either [M-H]^−^ or [M + FA-H]^−^. *m/z* values were mass corrected using the sodium formate injection during each LC–MS run. Full scans were collected from 15 to 1500 *m/z*. Mass spectral features were identified in MS-DIAL as [M-H]^−^, [M-2H]^2−^, or [M + FA-H]^−^ adducts with a minimum peak height of 500 amplitude. Mass spectral features are discrete signals in the LC–MS with defined RTs and *m/z*; however, there is not a one-to-one correlation between mass spectral features and compounds due to various adducts and isotope peaks [53]. Typical high-end LC–MS experiments identify >10 000 mass spectral features; however, the number of true metabolites is substantially fewer [53, 54]. Manual annotation was performed on these putative acylsugars, and those that were annotated were used for quantification. Acylsugars were quantified by summing all peak areas for identified acylsugars from LC–MS analysis performed in MS-DIAL. The total acylsugar peak area was corrected for the peak area of internal standard (telmisartan) and by tissue dry weight in grams. The resulting values were normalized to the most abundant extract for each organ type.

### MS/MS negative mode annotation of acylsugars

MS/MS was performed for a single acylsugar extraction from *Physalis nicandroides*. The same chromatography method and solvents were used as for acylsugar profiling. MS/MS was performed on a Waters Xevo-MRT operated in negative mode. A capillary voltage of 2 kV and a cone voltage of 40 V were used, with a desolvation temperature of 350°C and a source temperature of 150°C. The following ions were monitored 681.34, 695.35, 723.36, 737.37, and 751.38 with a collision energy ramp from 6 to 50 V, and dual lock mass correction was used monitoring 554.26 and 130.08 of leucine enkephalin.

### LC–MS positive and negative mode annotation of acylsugars

Acylsugar structures were inferred from *m/z* of intact adducts (either formate in negative mode or ammonium in positive mode) and fragmentation patterns in negative mode and positive mode (Supplementary Fig. S3) as previously described [20, 39]. Acylsugars were considered the same if the RT was within 0.05 minutes and mass-to-charge ratio (*m/z*) was within 0.1 *m/z*. Positive ion mode fragmentation was used to infer acyl chain positioning on the glucose or fructose monomer of the sucrose core, due to the typical cleavage of the glycosidic bond of the sucrose before ester bond cleavage of the acyl chains [39]. Acyl chains were annotated as described in Supplementary Fig. S4. Acylsugars with similar *m/z* yet differing RTs were considered to be different acylsugars, as this indicates the presence of isomers. Those with similar RTs, exact masses, and fragmentation patterns were considered to be the same acylsugar.

### Shannon entropy

Shannon entropy was calculated based on the presence or absence of all detected metabolites. Each detected metabolite feature was defined by its unique combination of its *m/z*, RT, and adduct type. To account for instrument variation and to align mass spectral features across samples, *m/z* and RT values were rounded to the nearest tenth prior to feature identification. Mass spectral features sharing identical rounded *m/z*, RT and adduct type values were treated as the same metabolite. Metabolite presence was determined for each sample, regardless of intensity. Shannon entropy was then computed via R using the formula:


$$ H=-{\sum}_{i=1}^n{p}_i\ {\mathit{\log}}_2{p}_i $$

where *p_i_* is the relative frequency of each mass spectral feature (in this case all equal due to just using absence and presence), and *n* is the total number of mass spectral features in the sample. Entropy values were then sorted by species and organ type, calculating mean entropy across samples. All data processing and entropy calculations were performed in RStudio using the readxl, dplyr, tidyr, ggplot2, multicompView [55].

### PCoA and HCA

Acylsugar annotation data were compiled into a binary absence (0) and presence (1) matrix, with rows representing species and columns representing unique annotations. Acylsugars with the same annotation but differing RTs were treated as unique annotations. Pairwise species dissimilarities were computed using Jaccard distance. Principal Coordinates Analysis was performed on the dissimilarity matrix, and the first two principal coordinate axes (PCoA1 and PCoA2) were visualized in a scatter plot. Each point represents a species, notated by the specific color and shape. These analyses were carried out using the readxl, dplyr, vegan, ape, and ggplot2 packages in R [55, 56].

HCA was performed with the same binary absence and presence matrix as was used for PCoA. Pairwise dissimilarity between species was computed using Jaccard distance, then the Jaccard distance matrix was projected into principal coordinate space. A new distance matrix was made from the top five PCoA axes, then hierarchical clustering performed using Ward's minimum variance method. The dendrogram was used to order species in a heatmap showing the absence (white) and presence (grey) of acylsugar annotations. Annotations retained their original order—sorted first by number of acylations then by *m/z.* These analyses were carried out using the readxl, dplyr, vegan, ComplexHeatmap, and circlize packages in R [57–59].

### Measures of phylogenetic signal

We estimated two measures of phylogenetic signal, namely Blomberg's K [41] for the continuously varying acylsugar abundance, and Fritz and Purvis's D (FPD, [42]) for binary acylation type (e.g., presence/absence of diacylation). We used a sample of 100 Bayesian time trees for Physalideae from Deanna et al. [1]. We pruned the trees to include only the species with trait and the trait data to include only taxa in the trees. For K, we used 1000 simulations to test for phylogenetic signal (K significantly greater than zero), and for D, we carried out 1000 permutations to estimate the probabilities of observing the data assuming no effect of phylogeny and of observing the data with evolution along the phylogeny following the Brownian motion process. We report mean, maximum, and minimum values for K and D for each trait across the 100 trees (Supplementary Tables S3 and S4), as well as the mean, maximum, and minimum *P* values (for K) and mean, maximum and minimum of P0 and P1 (for D). These analyses were carried out using the phytools 2.1–1[60] and caper 1.0.3 packages for R [61].

### GC–MS acyl chain analysis

Acylsugars were extracted as previously described using 3:3:2 isopropanol:acetonitrile:water with 0.1% formic acid in bulk from a subset of plants grown in a growth chamber. Because large amounts of tissue and solvent are required for bulk extracts, we were limited to analyzing plants that could be grown in growth chambers, and not those that were sampled in the field. Bulk extraction was performed from tissue collected from multiple plants of the same genotype. Extracts were dried to completeness in a centrivap. To cleave acyl chains from acylsugars and convert them into ethyl esters, 300 μl of 21% (v/v) sodium ethoxide was added to the dried bulk acylsugar extract. Samples were incubated at room temperature for 30 minutes with vortexing every 5 minutes. To isolate ethyl esters, 400 μl of hexane was added followed by vortexing and then 500 μl saturated sodium chloride was added to make a phase separation. The hexane layer was then transferred to a clean tube and additional sodium chloride was added. The hexane layer was moved to a fresh tube followed by sodium chloride. The final hexane layer was then moved to a 2-ml glass vial with a glass insert [21]. One microliter of sample was injected with a 10:1 split ratio onto a 60-m DB-5 ms column (Agilent Technologies). The initial oven temperature was 120°C, with an oven ramp rate of 6°C per minute until 300°C and a hold time of 8 minutes, and the inlet valve temperature was maintained at 280°C. The GC–MS was operated in full scan mode from a mass-to-charge ratio (*m/z*) of 50–650. Commercially available even chain, saturated fatty acid ethyl ester standards varying in length from C4 to C24 (Sigma) were used to aid in peak identification. Peaks absent in the even chain fatty acid ethyl ester standards (such as iC4, aiC5, iC5, and desaturated C5) were identified based on spectral matches to the NIST database.

### Sugar core analysis

Bulk extracts from a subset of *Physalis* species were dried to completion in a centrivap, then resuspended with 0.25 ml H_2_O and 1 ml chloroform. Aqueous and organic phases were allowed to separate, then the organic phase was transferred to a separate tube and dried at room temperature overnight. Once evaporated, the dried sample was resuspended in 100 μl of isopropanol. For saponification, 20 μl of sample was added to a tube with 200 μl methanol and 200 μl 3 N ammonium hydroxide. Sample was vortexed to mix then incubated at room temperature for 48 hours. Tubes were then dried to completion overnight in a centrivap. The dried extract was resuspended in 50 μl of a pyridine solution containing 15 mg/ml of methoxyamine HCl and incubated for 1 hour at 50°C, then 50 ml of *N,O*-Bis(trimethylsilyl)trifluoroacetamide +1% trimethylchlorosilane (BSTFA +1% TMCS; Supelco) was added and incubated for 1 hour at 50°C [62, 63].

Derivatized samples were injected into an Agilent 5977C GC–MS with authentic sugar standards (glucose and sucrose). One microliter of sample was injected with a 10:1 split ratio onto a 60-m DB-5 ms column (Agilent Technologies). The initial oven temperature was 120°C, with an oven ramp rate of 6°C per minute until 300°C and a hold time of 8 minutes, and the inlet valve temperature was maintained at 280°C. The GC–MS was operated in full-scan mode from a mass-to-charge ratio (*m/z*) of 50 to 650.

### ASAT identification and purification

ASATs in *Physalis* were identified using *P. pruinosa* genome. ASAT1-4 from *S. lycopersicum* were used to BLAST the *P. pruinosa* genome [31]. A total of 157 candidates were identified, which were filtered based on size, as ASATs are typically around 400 to 460 amino acids, as well as the presence of the HxxxD and DFGWG domains required for catalysis and substrate binding, respectively [64]. The 40 remaining candidates were grouped with biochemically characterized ASATs from other Solanaceae species [21–23] in a phylogenetic analysis using the Jukes-Cantor Distance model and neighbor-joining tree build method with 100 bootstrap replicates. A single candidate emerged based on the phylogenetic positioning with other AncASAT1s (Supplementary Fig. S16), as well as its high bit-score and *E* value.

Forward and reverse primers were designed based on *P. pruinosa* ASAT1, and were used to amplify ASAT1 from *P. pruinosa, P. cf. philadelphica,* and *P. philadelphica* genomic DNA, as ASATs typically do not have introns. Polymerase chain reaction (PCR) was performed using ThermoScientific Phusion DNA polymerase with a primer annealing temperature of 56°C, extension temperature of 72°C, and a total of 34 cycles. Primer pair used for gene amplification included the forward primer 5′-GCTAGCATGACTGGTGGACAGCAAATGGCTGCCTCAGCTCTA-3′ and reverse primer 5′-GCCGGATCTCAGTGGTGGTGGTGGTGGTGCTTATTCATCAACAAGTTTGAAT-3′. Amplified genes were purified by agarose gel electrophoresis and gel extraction using the Qiagen QIAquick gel extraction kit. ASAT candidate genes were cloned into pET28b expression vector through Gibson assembly according to manufacturer's instructions. Cloned gene sequences were confirmed via Sanger sequencing using vector and gene-specific primers and transformed into *Escherichia coli* Rosetta BL21 (DE3) cells for heterologous expression.

Cloned ASAT1s in Rosetta BL21 (DE3) cells were used for protein expression and purification. A 20-ml culture was grown overnight at 37°C, shaking at 200 rpm in LB media supplemented with 1% filter sterilized glucose, kanamycin at 50 μg/ml, and chloramphenicol at 25 μg/ml. Overnight culture was used to inoculate 3 L of fresh LB media, which was incubated at 37°C shaking at 200 rpm until an OD600 of 0.6 was reached. The cultures were then placed on ice for 15 minutes, then induced with 0.4 M IPTG. The cultures were then shaken at 18°C overnight. Cells were harvested by centrifugation at 5400 g for 10 minutes at 4°C. Pellets were resuspended in extraction buffer (25 mM HEPES pH 7.5, 50 mM KCl, 10% glycerol) and sonicated on ice for a total of 3 minutes, followed by a higher-speed centrifugation step for 30 minutes at 30 000 g. The supernatant was used for purification using Ni-NTA Agarose (Qiagen). Protein was washed stepwise with 10 ml each of increasing concentrations of imidazole (20, 40, and 60 mM), then with 5 ml of a final wash at 500 mM. The final wash was concentrated using 30 000 molecular weight cutoff centrifugal filters. Glycerol was added to a final concentration of about 50%, and then protein was stored at −20°C for subsequent analyses. Protein preps were analyzed by immunoblot analysis using a histidine-tag antibody (Supplementary Fig. S19).

### ASAT *in vitro* characterization

For functional characterization, purified ASAT1s were subjected to *in vitro* enzymatic assays. Enzymatic assays consisted of 250 μM commercially available acyl-CoAs of varying lengths, 2 mM sucrose, 250 mM sodium phosphate buffer pH 6, and 10 μl recombinantly purified enzyme. Assays were incubated for 1 hour at 30°C and stopped by addition of 60 μl 1:1 isopropanol and acetonitrile +0.1% formic acid and 5 μM telmisartan internal standard. Reactions were transferred to ice for 15 minutes, then centrifuged for 2 minutes. The supernatant was transferred to a glass vial with an insert and run on LC–MS equipped with a C18 column, with the same method used for acylsugar extracts (Supplementary Fig. S18). Activity was confirmed by the detection of the monoacylated sucrose using LC–MS. Specificity was quantified using peak area of the monoacylated product divided by the peak area of internal standard (telmisartan) and then normalized to the most abundant product. Two replicates of these assays were performed for each ASAT1.

## Supplementary Material

Web_Material_uhag177

## Data Availability

The code used in this study is available through GitHub https://github.com/lilliannowack. *Physalis* ASAT1s were deposited to NCBI with accession numbers *P. pruinosa* ASAT1 (PprASAT1): PV938311, *P. cf. philadelphica* ASAT1 (PcfphASAT1): PV938309, and *P. philadelphica* ASAT1(PphASAT1) PV938310.
